# Growth hormone in combination with leuprorelin in pubertal children with idiopathic short stature

**DOI:** 10.1530/EC-18-0137

**Published:** 2018-04-18

**Authors:** Imane Benabbad, Myriam Rosilio, Maité Tauber, Emmanuel Paris, Anne Paulsen, Lovisa Berggren, Hiren Patel, Jean-Claude Carel, the Phoenix Study Group

**Affiliations:** 1 Endocrinology and Diabetes UnitEli Lilly, Neuilly-sur-Seine, France; 2 Department EndocrineBone Diseases, Genetics, Obesity, and Gynecology Unit, Children’s Hospital, University Hospital, Toulouse, France; 3 BioClinicaLyon, France; 4 Assistance Publique-Hôpitaux de Paris (AP-HP)Hôpital Universitaire Robert-Debré, Department of Pediatric Endocrinology and Diabetology, and Centre de Référence des Maladies Endocriniennes Rares de la Croissance, Paris, France; 5 Eli Lilly and CompanyBad Homburg, Germany; 6 Eli Lilly and CompanyIndianapolis, Indiana, USA; 7 PROTECTINSERM, Université Paris Diderot, Sorbonne Paris Cité, Paris, France

**Keywords:** GH treatment, leuprorelin treatment, idiopathic short stature, growth, near-adult height

## Abstract

**Objective:**

There is a scarcity of data from randomised controlled trials on the association of growth hormone (GH) with gonadotrophin-releasing hormone agonists in idiopathic short stature (ISS), although this off-label use is common. We aimed to test whether delaying pubertal progression could increase near-adult height (NAH) in GH-treated patients with ISS.

**Methods:**

Patients with ISS at puberty onset were randomised to GH with leuprorelin (combination, *n* = 46) or GH alone (*n* = 45). NAH standard deviation score (SDS) was the primary outcome measure. The French regulatory authority requested premature discontinuation of study treatments after approximately 2.4 years; patients from France were followed for safety.

**Results:**

Mean (s.d.) baseline height SDS was −2.5 (0.5) in both groups, increasing at 2 years to −2.3 (0.6) with combination and −1.8 (0.7) with GH alone. NAH SDS was −1.8 (0.5) with combination (*n* = 19) and −1.9 (0.8) with GH alone (*n* = 16). Treatment-emergent adverse events and bone fractures occurred more frequently with combination than GH alone.

**Conclusion:**

Due to premature discontinuation of treatments, statistical comparison of NAH SDS between the two cohorts was not possible. During the first 2–3 years of treatment, patients treated with the combination grew more slowly than those receiving GH alone. However, mean NAH SDS was similar in the two groups. No new GH-related safety concerns were revealed. A potentially deleterious effect of combined treatment on bone fracture incidence was identified.

## Introduction

Children with height more than 2 standard deviations (s.d.) below the mean of a national reference population are considered to have idiopathic short stature (ISS) if the aetiology cannot be identified and disorders known to cause short stature have been excluded (1). Various studies have shown that growth hormone (GH) treatment of children with ISS can increase their growth velocity and, to a lesser extent, adult height (1, 2, 3, 4, 5, 6). The effect on height is variable and depends, among other factors, on dose and age at GH initiation (7, 8, 9, 10). GH treatment for ISS was approved in the United States in 2003 and has not been approved in the EU (4).

Children and adolescents with a diagnosis of ISS are often referred late, with GH treatment initiated when puberty has already started. Gonadotrophin-releasing hormone (GnRH) agonists administered alone for 2–3 years in this patient population delay puberty and epiphyseal closure but decrease growth velocity, resulting in no or a very limited effect on adult height (11, 12, 13). Delaying puberty using GnRH agonists has been suggested as a means to enhance GH efficacy, and some studies have reported positive effects on growth (14, 15). However, the studies have been small and have generally not included a relevant control group.

The present study was designed to investigate the treatment combination of GH and the GnRH agonist leuprorelin compared with GH alone in patients with ISS who were at puberty onset. It was hypothesised that the addition of leuprorelin for 2–3 years would delay progression of puberty and bone maturation, allowing prolonged efficacy of GH and increased adult or near-adult height (NAH). After commencement of the study, results communicated from the French cohort of the Santé Adulte GH Enfant (SAGhE) study suggested an increased risk during adulthood of mortality due to cerebrovascular complications and bone tumours among patients treated with GH as children for idiopathic isolated GH deficiency, ISS or born small for gestational age (16). The French Regulatory Agency, AFSSAPS, asked for GH treatment in this study to be stopped and, consequently, study drug treatments in the present study were terminated early. Patients in France continued to be followed for safety and evaluation of NAH in an amended protocol, and this report presents the available results from the study.

## Subjects and methods

### Study design and patients

This was a prospective, randomised, open-label, parallel-arm, comparative study designed to examine the safety and efficacy of GH with or without combination treatment with leuprorelin in pubertal children with ISS (ClinicalTrials.gov: Nbib355030). The study was carried out between June 2006 and July 2015 in study centres in France and the Netherlands. The study was approved by Comité de Protection des Personnes Ile-de-France III Hôpital Tarnier-Cochin 89, rue d’Assas, 75006 Paris. The study included a treatment period and a subsequent post-treatment safety follow-up period after treatment discontinuation was requested in 2011 (Supplementary Fig. 1, see section on supplementary data given at the end of this article).

Patients enrolled were either females aged ≥8 years and ≤12 years 3 months, with bone age ≤12.0 years or males aged ≥9 years and ≤14 years 3 months, with bone age ≤14.0 years. Pubertal status had to be Tanner stage B2 or B3 for girls and, for boys, either Tanner stage G2 or G3, testicular length ≥30 mm and <40 mm or testicular volume ≥4 mL and <12 mL. All patients had a diagnosis of ISS, defined as height standard deviation score (SDS) ≤−2.5 based on national references or height SDS ≤−2 and predicted adult height SDS ≤−2.5 based on the Bayley–Pinneau method (17) and confirmed by a centralised bone age reading. Patients were excluded if they had GH deficiency (defined as peak GH ≤20 IU/L in each of two stimulation tests), had insulin-like growth factor (IGF)-I SDS >3, were born small for gestational age, had a diagnosed chromosomal anomaly, had reached menarche, had any significant concomitant disease that was likely to interfere with growth or presented with a lumbar spine bone mineral density (BMD) <−2 SDS from height- and age-adjusted *Z*-score. Patients who had previously been or were currently being treated with any drug that could directly influence growth or had a contraindication to treatment with either GH or a GnRH agonist were also excluded.

The clinical trial protocol was approved by all appropriate local ethics review committees, and the protocol adhered to the applicable regulatory requirements in the participating countries, with appropriate institutional review board approvals. For both the original study period (treatment period) and the safety follow-up period, written informed consent for data collection, processing and publication was provided by the patient, parents or a legal guardian of each child, in accordance with national and local regulations. Both periods of the study were performed according to the ethical principles of the Declaration of Helsinki.

### Randomisation and study treatments

Patients were randomised on a 1:1 basis to treatment with GH (Humatrope, Eli Lilly and Company) with or without leuprorelin 3-month depot (Enantone, Takeda, Puteaux, France). The initial protocol included a GH-untreated control group, but this group was stopped because of recruitment and control arm patient retention difficulties and eligible patients were re-randomised to either GH plus leuprorelin or GH alone. GH was administered daily by subcutaneous injection at a dose of 0.05 mg/kg, which was planned to be continued until NAH was reached. GH dose was decreased by 25% if IGF-I SDS increased to >3.0 or if IGF-I SDS was >0.5 at the same time as IGF-binding protein (IGFBP)-3 SDS was <−0.5. Leuprorelin was given by subcutaneous or intramuscular injection at a dose of 11.25 mg every 3 months for a planned minimum of 2 years and a maximum of 3 years or until patients reached a chronological age of 13 years for girls or 15 years for boys. Duration of leuprorelin treatment was calculated as the time from the first injection to 3 months after the last injection.

After cessation of study drugs at the request of the French regulatory authority, and following a protocol amendment, all patients in the study in France were offered participation in a follow-up study, without study drug treatment, for safety assessments until they reached NAH. Patients in the Netherlands were invited to join the Genetics and Neuroendocrinology of Short Stature International Study (GeNeSIS; ClinicalTrials.gov: Nbib1088412) for follow-up. The safety follow-up data in the present report include only the patients in France, not the patients from the Netherlands.

### Study evaluations

Baseline data collected included demographics, medical history, laboratory values, bone age, gonadotropin levels and pubertal status. Other clinical variables measured at baseline included height, weight, parental heights (for calculation of target height (sex-adjusted average of parental heights)) and body composition. Height was determined barefoot using a wall-mounted Harpenden stadiometer. Bone age was determined from a centralised reading of an X-radiograph of the left hand and wrist, using Greulich and Pyle standards (18). Predicted adult height was calculated using the Bayley–Pinneau method on the basis of actual height and bone age (17). Follicle-stimulating hormone (FSH) and luteinising hormone (LH) levels were measured before and after stimulation with LH-releasing hormone (LHRH).

It was planned that patients would attend study visits at 3 months, 6 months and then every 6 months up to 5 years. Height, weight and pubertal stage were to be documented at each study visit and patients were to be followed up until they were considered to have reached NAH. Criteria for NAH were bone age ≥15 years for girls or ≥17 years for boys, and growth ≤1 cm in the previous 6 months. NAH SDS was calculated using chronological age at the time of the relevant visit.

Safety analysis was based on laboratory assessments, including IGF-I, IGFBP-3, fasting blood glucose, insulin, glycated haemoglobin (HbA1c), lipids, free thyroxine (T4) and thyroid-stimulating hormone (TSH) levels, body composition changes, vital signs and reported adverse events. Body composition was determined at baseline and yearly intervals using dual-energy X-ray absorptiometry and included total body and lumbar spine bone mineral content (BMC) and BMD, total spine bone mineral apparent density (BMAD) (19), total body fat mass and lean body mass. For patients treated with leuprorelin, if BMD decreased from baseline by >5% a second measurement was scheduled for 6 months later; if the second measurement showed a decrease >5%, confirmed by central laboratory reading, leuprorelin was discontinued, but the patient could continue with GH alone. For additional sensitivity and patient safety, leuprorelin treatment was discontinued if BMD *Z*-score decreased to <−2 SDS at any time. Blood pressure was evaluated according to reported age- and sex-matched reference values (20). Severity and relationship of adverse events to study drug treatment were determined by the attending physician. Adverse events were classified as treatment emergent if they first occurred or worsened during study drug treatment. Serious adverse events were defined as any event that resulted in death, hospitalisation, persistent or significant disability or congenital anomaly in the offspring of a treated patient, were considered life threatening or were significant for other reason in the opinion of the investigator. All adverse events were categorised according to the Medical Dictionary for Regulatory Authorities (MedDRA, version 18.0).

### Statistical analyses

The initial primary objective was to compare the NAH SDS of patients treated with GH plus leuprorelin with that of patients treated with GH alone, in patients with ISS. A sample size of 44 patients per treatment group was estimated to provide 80% power of detecting a statistically significant difference of 0.55 for NAH SDS in a two-sided test at a 5% significance level. Because study drug treatment was terminated early, the objectives of the study could not be met and no formal analyses were performed. The main objective for the safety follow-up period was to monitor the short- and long-term safety of GH therapy, with or without leuprorelin.

Secondary objectives were to describe the annual height velocity and height SDS, gains in these variables from baseline to NAH and differences between NAH SDS and both target height SDS and baseline predicted height SDS in the two treatment groups. Descriptive statistics were calculated for observed data relating to auxology and safety variables at each study visit, including change from baseline at each post-baseline visit. All analyses were performed using only data from patients who provided measurements at the relevant visit. Descriptive analyses were used to assess the clinical progression of puberty and changes in body composition and bone from baseline to study endpoint in the two treatment groups.

## Results

### Disposition and study drug exposure

**Figure 1:**
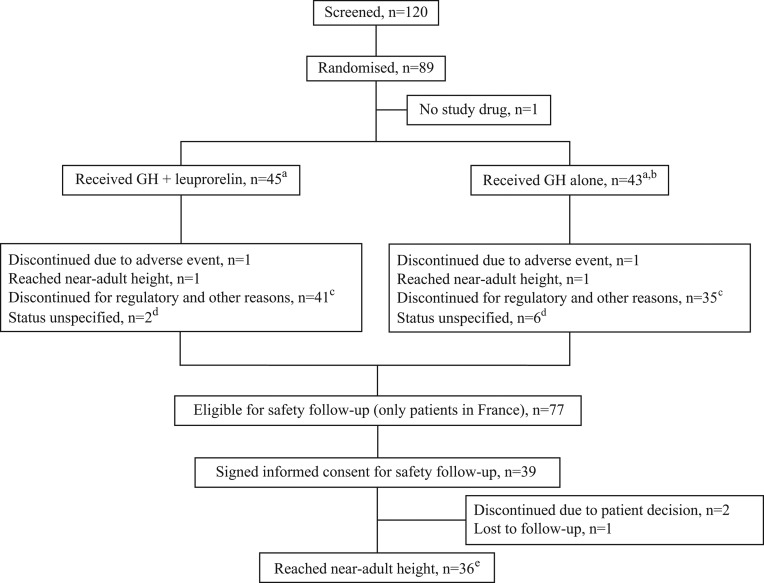
Patient disposition during the study. ^a^One patient randomised to growth hormone (GH) alone also received leuprorelin; this patient was evaluated in the GH alone group for auxology and in the GH plus leuprorelin group for safety. ^b^An additional three patients from a previous control group who did not meet entry criteria received GH treatment for ethical reasons and hence were included for safety analyses only. ^c^Study treatment stopped at request of the French Agency for the Safety of Medicines and Health Products. ^d^Study treatment stopped and the relevant study discontinuation report forms were not completed by the investigator. ^e^Included one patient from each treatment group who reached near-adult height prior to study drug termination.

A total of 89 patients were randomised to study treatment (Fig. 1), of whom 45 were randomised to GH plus leuprorelin and 44 randomised to GH alone; one patient randomised to GH alone violated an exclusion criterion and did not receive study treatment. The 88 treated patients (France 77 patients, Netherlands 11 patients) comprised the population analysed according to randomisation group for auxology data. The safety population for the treatment period comprised 46 patients who received GH plus leuprorelin and 45 who received GH alone; this included one patient randomised to GH alone but inadvertently also received leuprorelin and three patients from a previous control group who received GH but were ineligible for analysis of auxology data. Following treatment discontinuation, 39 patients signed informed consent for safety follow-up. NAH information was available for 36 patients overall in the study, with 35 having evaluable auxology information.

Mean (s.d.) duration of GH treatment for the 46 patients who received GH plus leuprorelin was 28.9 (10.9) months (range 1.7–49.2) and for the 45 patients who received GH alone was 29.9 (10.7) months (range 3.1–51.3). Mean dose of GH at 3 and 12 months were 46 (8) and 46 (7) µg/kg/day in the GH plus leuprorelin group and 48 (4) and 46 (4) µg/kg/day in the GH alone group. Mean exposure to leuprorelin in the combination group was 20.9 (6.4) months (range 3.0–33.1).

### Effects on auxology

For patients who were included in the auxology analysis, there were no evident baseline differences between the groups in chronological age, bone age or height parameters (Table 1).

**Table 1:** Patient demographics and characteristics at baseline for patients randomised to treatment with growth hormone with or without combined leuprorelin treatment.

Characteristics	GH + leuprorelin (*n* = 45)	GH alone (*n* = 43)
Chronological age (years)	12.1 (1.4)	12.1 (1.3)
Female	26/45	20/43
Ethnic origin, Caucasian	36/45	40/43
Bone age (years)	10.8 (1.6)	11.0 (1.5)
Bone age delay (months)	15.2 (15.6)	12.6 (12.3)
Body mass index (kg/m^2^)	16.8 (2.0)	16.3 (1.5)
Height SDS	−2.5 (0.5)	−2.5 (0.5)
Height velocity (cm/year)	7.8 (6.9)	7.4 (6.0)
Target height SDS	−1.1 (0.9)	−1.0 (0.8)
Predicted height SDS	−2.7 (0.8)	−2.6 (0.9)
Target height SDS – predicted height SDS	1.5 (1.0)	1.7 (1.2)
IGF-I (µg/L)	177 (128:246)	164 (129:216)
IGF-I SDS	−1.26 (−1.90:−0.44)	−1.51 (−2.68:−0.59)
IGF binding protein-3 (mg/L)	3.23 (2.57:3.77)	2.90 (2.36:3.21)
IGF binding protein-3 SDS	0.02 (−0.93:0.51)	−0.41 (−1.24:0.09)

Data show mean (s.d.) or number of patients as a proportion of the total with available data, except for IGF concentrations shown as median (25th percentile:75th percentile).

GH, growth hormone; IGF, insulin-like growth factor; s.d., standard deviation; SDS, SD score.

**Figure 2:**
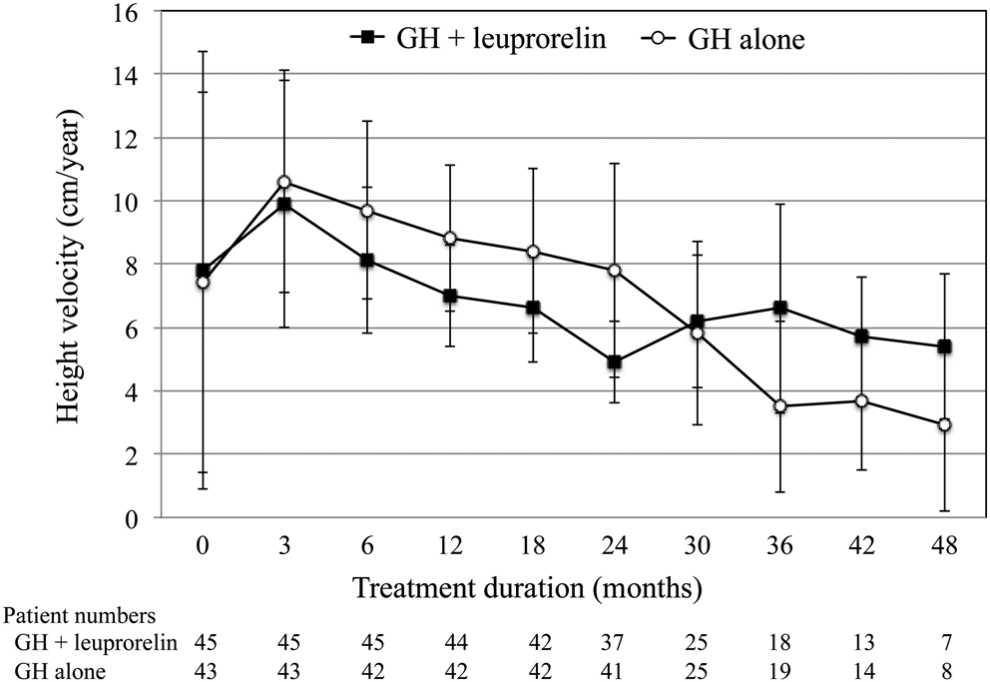
Height velocity over time for patients with idiopathic short stature treated with growth hormone (GH) with or without leuprorelin. Data shown as mean ± standard deviation (s.d.). The mean (s.d.) duration of leuprorelin treatment in the combination group was 20.9 (6.4) months.

**Figure 3:**
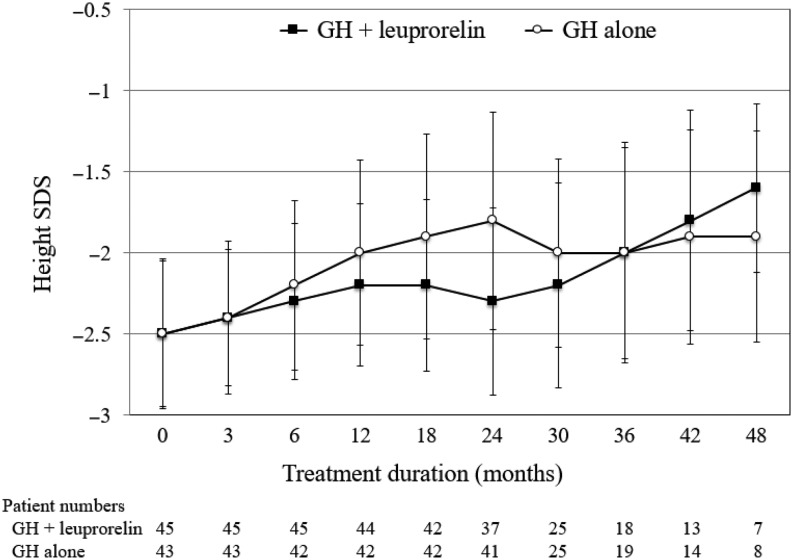
Height standard deviation (s.d.) score (SDS) over time for patients with idiopathic short stature treated with growth hormone (GH) with or without leuprorelin. Data show mean ± s.d. The mean (s.d.) duration of leuprorelin treatment in the combination group was 20.9 (6.4) months.

Baseline mean (s.d.) height velocity (Fig. 2) was 7.8 (6.9) and 7.4 (6.0) cm/year in the GH plus leuprorelin group and the GH alone group, respectively, and was 8.1 (2.3) and 9.7 (2.8) cm/year, respectively, at 6 months. After 2 years, mean height velocity was 4.9 (1.3) and 7.8 (3.4) cm/year in the two groups, respectively. Mean height velocity SDS showed the same trend. Mean height SDS (Fig. 3) increased in the GH alone group and was lower in the GH plus leuprorelin group for the first 2 years; when leuprorelin was stopped, a catch-up of mean height SDS was seen, to a level that appeared similar to that of the GH alone group.

Mean (s.d.) bone age delay at baseline was 15.2 (15.6) months in the GH plus leuprorelin group, increasing to 21.4 (14.6) months after 2 years of treatment. In the GH alone group, bone age delay was 12.6 (12.3) months at baseline and decreased to 10.7 (14.0) months after 2 years of treatment.

NAH was available for 19 patients from the GH plus leuprorelin group and 16 patients from the GH alone group (Table 2). The mean (s.d.) height SDS gain from baseline to the time of NAH measurement was 0.6 (0.6) in the combination group and 0.6 (0.7) in the GH alone group. NAH SDS within the normal range (>−2 to <2) was achieved by 12/19 (63%) patients in the GH plus leuprorelin group and 11/16 (69%) patients in the GH alone group.

**Table 2:** Height information for patients who reached near-adult height.

	GH + leuprorelin (*n* = 19)	GH only (*n* = 16)
Near-adult height SDS	−1.8 (0.5)	−1.9 (0.8)
Near-adult height SDS gain from baseline	0.6 (0.6)	0.6 (0.7)
Near-adult height – predicted height (cm)	5.0 (7.2)^a^	6.0 (4.0)
Near-adult height SDS – predicted height SDS	1.1 (1.0)^a^	1.2 (0.7)
Near-adult height – target height (cm)	−3.7 (5.1)^b^	−7.1 (4.5)^a^
Near-adult height SDS – target height SDS	−0.6 (0.9)^b^	−1.2 (0.8)^a^
Proportion with near-adult height SDS >−2 to <2	12/19	11/16

Data show mean (s.d.) or number of patients as a proportion of the total with available data.

^a^Data missing for one patient; ^b^data missing for three patients.

GH, growth hormone; SDS, standard deviation score.

### Safety information

No deaths were reported during the study. During the treatment phase of the study, serious adverse events were reported for 12/46 (26.1%) patients in the GH plus leuprorelin group and 7/45 (15.6%) in the GH alone group. One serious adverse event was reported by the investigator to be related to study drug, which was a transient event of migraine in a 12-year-old girl in the GH plus leuprorelin group. A 12-year-old girl in the GH plus leuprorelin group discontinued GH treatment approximately 7 weeks after initiation due to histiocytosis, although leuprorelin was continued for an unspecified period. Magnetic resonance imaging of the 5th and 6th thoracic vertebrae and biopsy confirmed histiocytosis, which was thought to probably have been present before study drug administration and was considered by the investigator to be unrelated to treatment. A 13-year-old boy in the GH alone group experienced impaired glucose tolerance after 3 years of treatment, which was considered to be treatment related; GH was discontinued, and the event was reported to be resolved approximately 13.5 months later.

During the treatment phase, treatment-emergent adverse events were reported for 42/46 (91.5%) patients in the GH plus leuprorelin group and 38/45 (84.4%) in the GH alone group. The system organ classes with treatment-emergent adverse events most frequently reported were infections and infestations (combination group 33/46 (71.7%) and GH alone group 24/45 (53.3%)), nervous system disorders (21/46 (45.7%) and 17/45 (37.8%)) and musculoskeletal and connective tissue disorders (19/46 (41.3%) and 14/45 (31.1%)). The most frequently reported preferred term events were headache (combination group 21/46 (45.7%) and GH alone group 16/45 (35.6%)), nasopharyngitis (19/46 (41.3%) and 8/45 (17.8%)) and influenza (15/46 (32.6%) and 9/45 (20.0%)). The treatment-emergent events were considered related to study drug for 12/46 (26.1%) patients in the GH plus leuprorelin group and 6/45 (13.3%) in the GH alone group. All of the related events were considered to be expected based on the known safety profile of GH, except for the one case of migraine reported as a serious adverse event. Treatment-emergent scoliosis was reported for four patients in the GH plus leuprorelin group and one patient in the GH alone group; the events were considered related to treatment for two patients in the GH plus leuprorelin group.

During the safety follow-up period, serious adverse events were reported for two patients (renal colic and appendicitis) who had previously been treated with GH plus leuprorelin. Overall, 20 of 39 patients (51.3%) reported an adverse event, of which one event of scoliosis in a patient previously treated with GH plus leuprorelin was considered related to study drug treatment.

**Figure 4:**
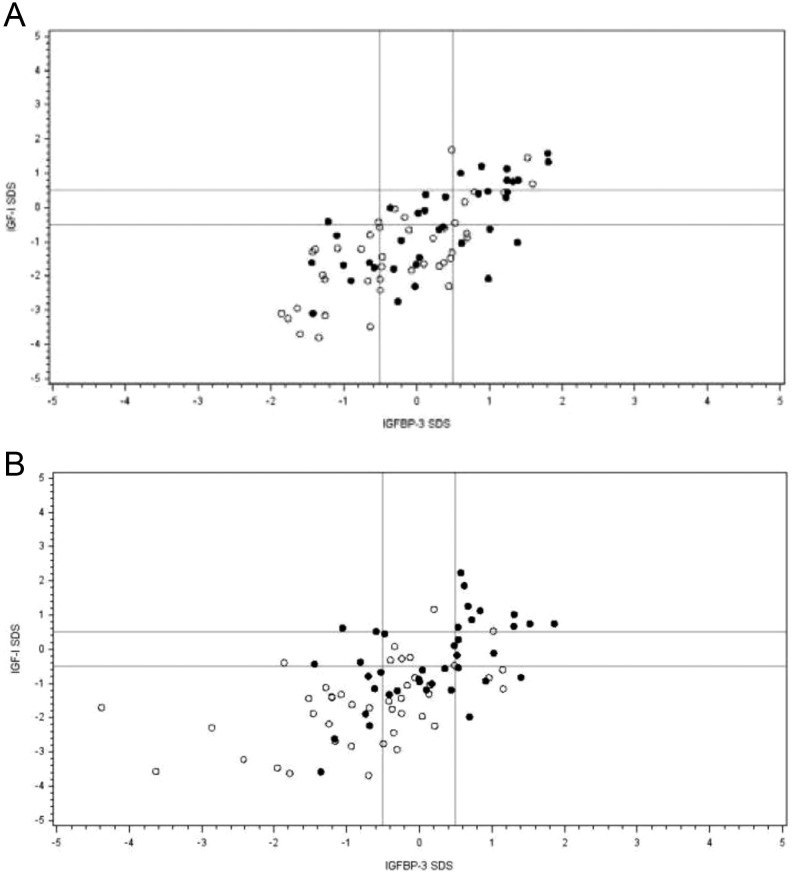
Scatter plots of insulin-like growth factor (IGF)-I standard deviation score (SDS) vs IGF-binding protein (IGFBP)-3 SDS at baseline (open circles) and after 24 months (filled circles) in patients with idiopathic short stature treated with either growth hormone (GH) plus leuprorelin (A) or GH alone (B). Horizontal lines show IGF-I SDS ± 0.5 and vertical lines show IGFBP-3 ± 0.5.

At baseline, median IGF-I SDS was −1.26 and −1.51 in the GH plus leuprorelin and GH alone groups, respectively, and median IGFBP-3 SDS was 0.02 and −0.41, respectively (Supplementary Table 1). Scatter plots of IGF-I SDS vs IGFBP-3 SDS at baseline and after 2 years of treatment are shown in Fig. 4. For patients with NAH values, median IGF-I SDS in the groups previously treated with GH plus leuprorelin (*n* = 5) and GH alone (*n* = 10) was −1.25 and −1.02, respectively, and median IGFBP-3 was −0.23 and −0.54, respectively. No patients in either treatment group had an IGF-I SDS >2 and IGFBP-3 SDS <−2 concomitantly at any time during the study.

No adverse events of hypertension or other events related to elevated blood pressure were reported during the treatment phase or the safety follow-up of the study. Over the entire study period, elevated systolic and diastolic blood pressure were noted for 25 and 16 patients, respectively, which was transient in almost all cases and with no obvious difference between the treatment groups. During safety follow-up, transient elevations in systolic blood pressure were noted for eight patients and diastolic blood pressure for two patients, with no evident difference between treatment groups.

### Bone health and body composition

**Figure 5:**
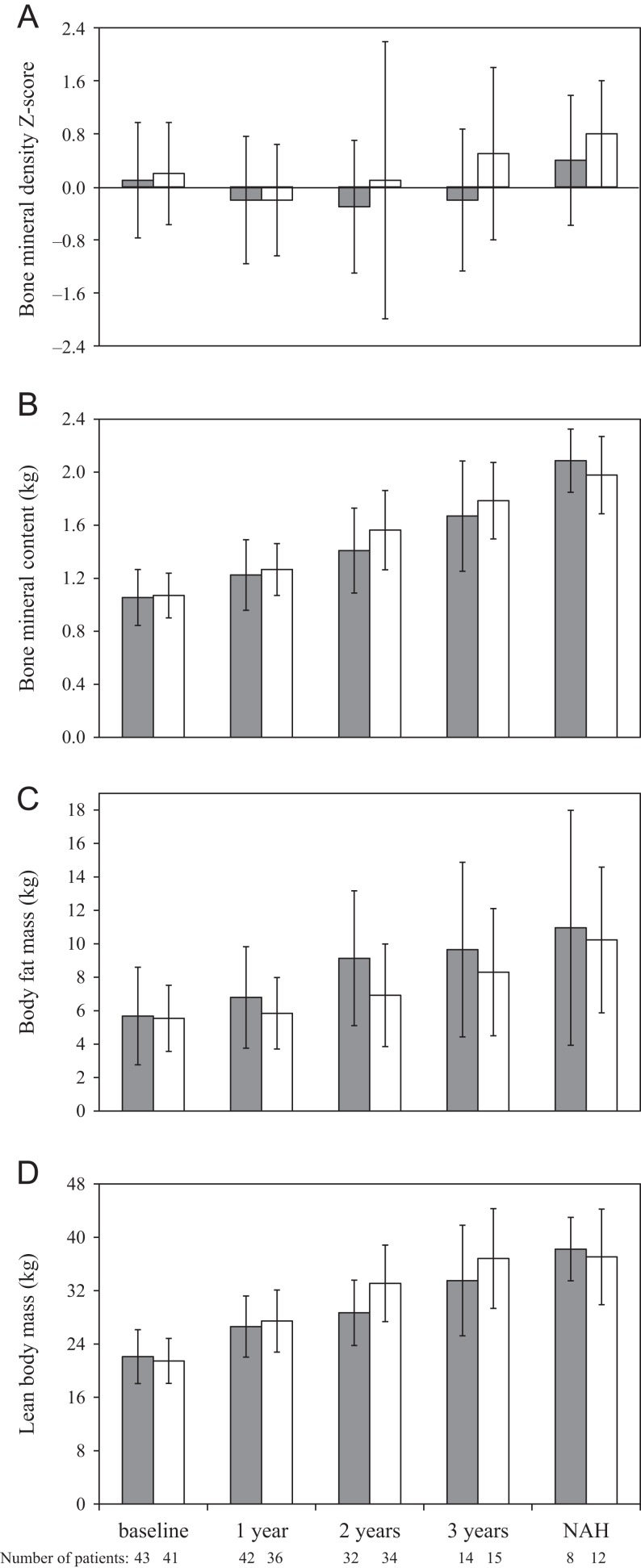
Total body bone mineral density *Z*-score, bone mineral content, fat mass and lean body mass at baseline and during treatment and at near-adult height (NAH) with either growth hormone (GH) plus leuprorelin (shaded bars) or GH alone (white bars). Data are shown as mean ± standard deviation (s.d.). Mean (s.d.) duration of leuprorelin treatment was 20.9 (6.4) months for the safety population.

Total spine BMAD mean (s.d.) at baseline was 0.120 (0.023) and 0.121 (0.023) g/cm^3^ in the GH plus leuprorelin and GH alone groups, respectively, and after 2 years of treatment was 0.126 (0.023) and 0.134 (0.025) g/cm^3^, respectively. Total body BMD *Z*-score (Fig. 5) at baseline was 0.1 (0.9) and 0.2 (0.8), respectively, and after 2 years was −0.3 (1.0) and 0.1 (2.1), respectively. In the GH alone group, BMC, fat mass and lean body mass increased gradually during the 3 years of treatment, while BMD *Z*-score appeared stable during the first 2 years of treatment and increased in the third year (Fig. 5). In the GH plus leuprorelin group, lesser increases in BMC and lean body mass, with a larger increase in fat mass, were observed. In the GH plus leuprorelin group, BMD *Z*-score tended to decrease at year 2 and return towards baseline values at year 3. Body composition data were available at NAH for eight patients previously treated with GH plus leuprorelin and 12 patients with GH alone, and showed similar levels of BMC (2.09 and 1.98 kg, respectively), fat mass (11.0 and 10.2 kg, respectively) and lean body mass (38.2 and 37.1 kg, respectively) (Fig. 5).

Bone fractures during treatment occurred in seven patients in the GH plus leuprorelin group and in three patients in the GH alone group. The fractures were classified as serious adverse events for four of the patients in the combination group, two of whom were the only study participants to have BMD *Z*-scores <−2 SDS (one at year 2 with improvement in BMD after stopping leuprorelin, one at year 1 with no further information) and who were discontinued from leuprorelin treatment. No bone fractures were reported during the safety follow-up period.

### Gonadotropins and pubertal development

Mean (s.d.) LHRH-stimulated FSH concentration decreased during leuprorelin treatment from 7.9 (4.3) U/L at baseline to 3.1 (3.0) U/L at 2 years of treatment; mean stimulated LH concentrations also decreased from 14.6 (8.0) U/L at baseline to 4.4 (6.6) U/L at 2 years. After leuprorelin was stopped, monitoring of 18 patients in the GH plus leuprorelin group at the 30-month visit showed LHRH-stimulated FSH and LH concentrations were 8.1 (6.1) U/L and 14.0 (11.1) U/L, respectively.

At the start of treatment, the majority of patients in both treatment groups were at pubertal stage G2 for boys (27/42 (64%) overall) or B2 for girls (30/46 (65%) overall; Supplementary Table 2). Pubertal stage remained at G1/G2 or B1/B2 for the majority of patients through the 2 years of GH plus leuprorelin treatment (boys 9/15 (60%), girls 18/21 (86%)). When leuprorelin was stopped, progression of pubertal stage continued and, at 3 years, the majority of patients were at G4 or B3/B4. In the GH alone group, pubertal stage progressed and the majority of patients were at stage G4 or B4 at 3 years.

## Discussion

This study was designed to evaluate the benefits and risks of GH combined with a GnRH agonist (leuprorelin) compared with GH alone in patients with ISS who were initiating treatment at the start of puberty. The hypothesis was that slowing pubertal progression would increase the duration of the growth spurt and allow GH to continue acting for a longer period, ultimately providing an increased adult height. Following the French regulatory authority request to stop GH treatment in this study, both study drugs were discontinued for all patients. As a result, the primary endpoint could not be evaluated, limiting any conclusions. Nevertheless, patients received a mean of 21 months of leuprorelin and 29 months of GH, compared with the planned exposure of 24–36 months of leuprorelin and prolonged GH treatment until attainment of NAH. Patients in France were invited to participate in a long-term safety follow-up, and patients in the Netherlands were encouraged to participate in the GeNeSIS observational programme. The results from the patients in France who entered the safety follow-up presented no new safety concerns regarding GH administration, but potential safety concerns with the combination therapy were identified. The limited auxology results indicated that, during leuprorelin treatment, the GH-induced gain in height was reduced, but the height gain increased after leuprorelin was stopped. Thus, the overall height gains after GH treatment were similar for patients treated with or without leuprorelin, and the height SDS gains for patients who reached NAH during the study were comparable. These results raise questions concerning the efficacy and safety of combined GH and GnRH agonist treatment.

As expected, leuprorelin decreased FSH and LH release, producing a delay in progression of puberty, consistent with the known effects of GnRH agonists (21, 22). The effects on gonadotrophins and puberty were reversible when leuprorelin was stopped, and growth of the patients re-accelerated. A similar decrease in height SDS gain is observed when GnRH agonists alone are used during puberty (23), and our results confirm the role of sex steroids in statural growth at puberty, a role that cannot be replaced by GH treatment. After cessation of GnRH agonist treatment, growth resumed. For patients who achieved NAH during the study, the height SDS was comparable between treatment groups, with similar proportions of patients in each group achieving NAH SDS within the normal range. Thus, in the conditions used in our study, where the study protocol was not fulfilled because of premature cessation of treatment, no firm conclusion can be drawn. However, there seemed to be no additional increase in NAH with the combination, compared with GH alone. Similarly, when GnRH agonists have been used outside precocious puberty, with or without concomitant GH, growth increase has been shown very inconsistently (11, 24, 25, 26, 27).

One important finding of our study was the reduced mean total body BMC increase in the GH plus leuprorelin group after 2 years of treatment. This was consistent with reported reductions in bone accretion with GnRH agonists (12, 27, 28). Similarly, during GH plus leuprorelin treatment, fat mass appeared to increase more, whereas lean body mass appeared to increase less than with GH alone. This was consistent with a study that showed that GH treatment of adolescents with ISS was associated with decreased fat mass and increased lean body mass (29). Another study showed that combined GnRH agonist plus GH treatment increased fat mass gain and decreased lean body mass gain vs GH alone in short children born small for gestational age (30). Therefore, our results are consistent with published studies and suggest that the expected effects of leuprorelin on body composition are not counterbalanced by GH when combined treatment is administered for a limited time and GH not pursued until adult height.

The adverse events reported in the study were consistent with the safety profile of GH in patients with ISS, and IGF-I was not increased excessively (1, 3, 8, 31, 32). Because GnRH agonist therapy is associated with decreased BMD, concerns have been raised concerning potential fractures due to the reduced bone accretion (12). Indeed, in our study, bone fractures occurred more frequently in the combined GH plus leuprorelin group than the GH alone group, and the fractures were associated with an abnormally low BMD in two cases. Concerns raised by results from the SAGhE study led investigators to closely monitor blood pressure, but there were no reports of hypertension or other events related to elevated blood pressure.

Our study has several limitations that need to be highlighted. First, as discussed, the premature interruption of treatments at the request of French authorities prevented evaluation of the effects of treatments as planned in the protocol. Nevertheless, the exposure to treatments was around 30 months for GH and 21 months for leuprorelin. However, both GH and leuprorelin were stopped together, whereas the protocol stated that for the leuprorelin plus GH group leuprorelin treatment should stop first and GH treatment continue for a further 1–2 years, resulting in the combination arm receiving a suboptimal treatment regimen. Second, we could only follow 36 patients to NAH, compared with the 88 patients randomised (41%) and 77 patients (47%) eligible for long-term follow-up in France and reported here. However, the two groups followed to NAH were well balanced in terms of characteristics. Lastly, although the initial protocol included an untreated control group, the study did not evaluate the effect of GH vs no treatment in patients with ISS as the control group was discontinued due to recruitment and control arm patient retention difficulties.

## Conclusion

Premature discontinuation of both study treatments upon request from the French regulatory authority meant that formal comparison of the effects of combination treatment vs GH alone in children with ISS was not possible. However, in these young patients with ISS treated near the onset of puberty, short-term GH-induced height SDS gain seemed to be reduced by the addition of leuprorelin to the treatment regimen, although some catch-up growth was observed after cessation of leuprorelin. Looking at the longer-term effects of combination therapy on NAH, no difference was observed in terms of growth gains from the addition of leuprorelin to GH therapy. The safety follow-up after discontinuation of GH revealed no new safety concerns relating to GH treatment. A potentially deleterious effect of combination treatment on bone fracture incidence was identified.

## Supplementary Material

Supporting Figure 1

Supporting Table 1

Supporting Table 2

## Declaration of interest

I B, L B, H P and M R are employees of Eli Lilly and Co. J-C C, M T and E P have received honoraria from Eli Lilly and Co. for advisory board meetings at early stages of the project. A P has nothing to declare.

## Funding

Eli Lilly and Company.

## Author contribution statement

M R, J-C C, MT and I B conceived and designed the study. J-C C, M T, E P, L B, H P and A P performed the clinical measurements or contributed to data analysis. All authors contributed to the writing of the manuscript and agree with the final manuscript and conclusions.
