# Supplementary figures and images for: Growth hormone in combination with leuprorelin in pubertal children with idiopathic short stature
Source: Endocr Connect. 2018 Apr 18;7(5):708–18. doi: 10.1530/EC-18-0137 (PMC5952247; doi:10.1530/EC-18-0137)

Supplemental Fig. 1

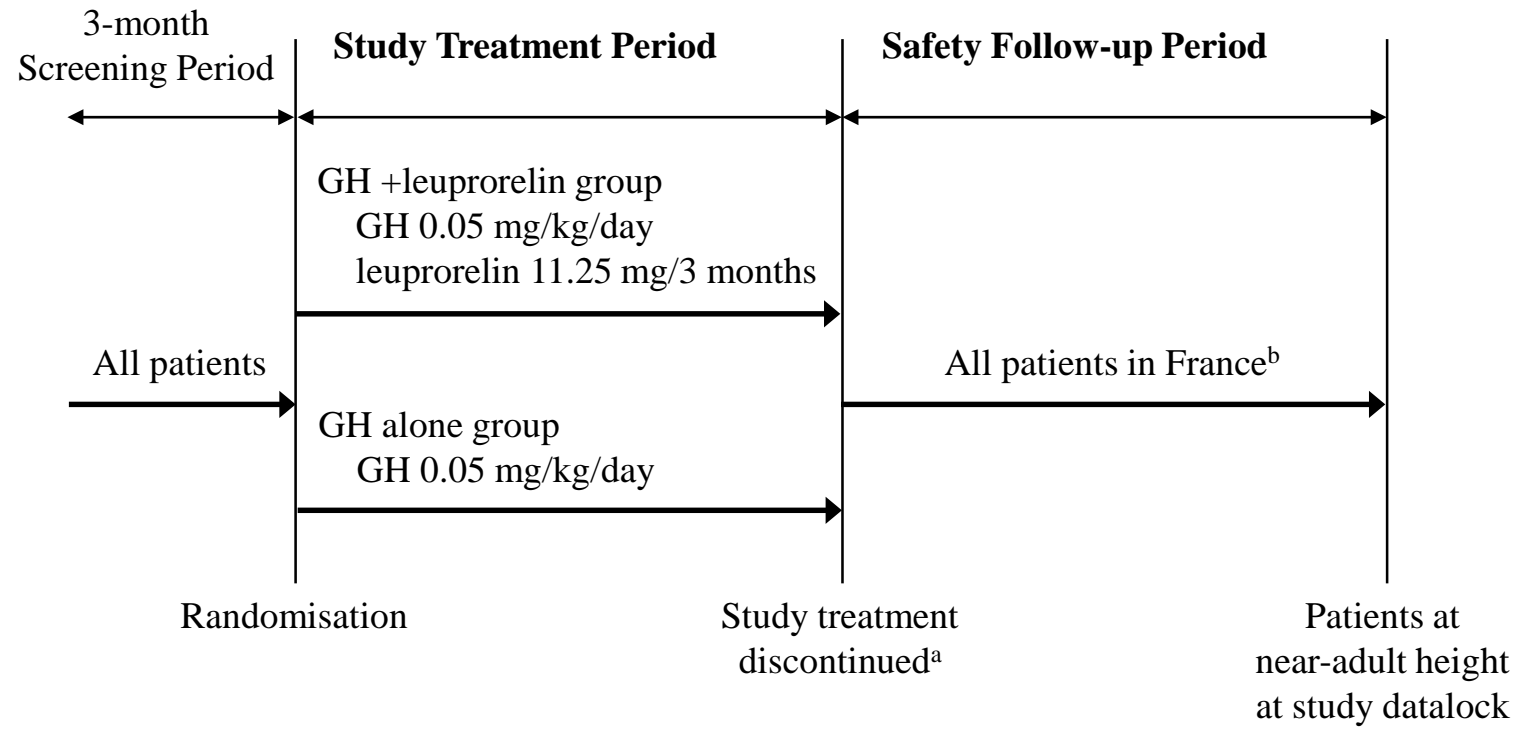

Supplement: Supporting Figure 1 [file ec-7-708-s001.pdf]
