# Supplementary material for: Growth hormone in combination with leuprorelin in pubertal children with idiopathic short stature
Source: Endocr Connect. 2018 Apr 18;7(5):708–18. doi: 10.1530/EC-18-0137 (PMC5952247; doi:10.1530/EC-18-0137)
Supplement: Supporting Table 1 [file ec-7-708-t001.pdf]

Supplemental Table 1 IGF-I SDS and IGFBP-3 SDS at baseline and by duration of treatment with GH with or without leuprorelin.

	GH + leuprorelin			GH alone		
Treatment duration	n	IGF-I SDS	IGFBP-3 SDS	n	IGF-I SDS	IGFBP-3 SDS
baseline	40	−1.26 (−1.90 : −0.44)	0.02 (−0.93 : 0.51)	41	−1.51 (−2.68 : −0.59)	−0.41 (−1.24 : 0.09)
6 months	38	0.25 (−0.38 : 0.980	0.61 (0.15 : 0.97)	37	0.15 (−1.09 : 0.89)	0.34 (−0.18 : 1.11)
12 months	34	0.10 (−0.70 : 0.84)	0.53 (0.04 : 1.02)	35	0.07 (−1.06 : 0.39)	0.49 (−0.06 : 1.20)
18 months	29	−0.14 (−1.03 : 0.83)	0.72 (−0.04 : 1.34)	28	−0.15 (−0.98 : 0.29)	0.36 (−0.26 : 0.84)
24 months	20	−0.36 (−1.42 : 0.62)	0.74 (−0.12 : 1.24)	25	−0.54 (−1.20/0.63)	0.36 (−1.03 : 0.84)
30 months	14	−0.39 (−1.25 : 0.51)	0.23 (−0.07 : 0.68)	17	0.39 (−0.51 : 0.96)	0.65 (0.04 : 1.32)
36 months	9	0.29 (0.25 : 0.63)	0.86 (0.32 : 1.21)	16	−0.12 (−0.39 : 1.06)	0.74 (0.12 : 0.99)

Data show median (25th percentile : 75th percentile); insufficient numbers of patients had data beyond 3 years of treatment.

n, total number of patients with data available; IGF-I, insulin-like growth factor-I; IGFBP-3, IGF binding protein-3; SDS, standard deviation score.
