# Supplementary material for: Growth hormone in combination with leuprorelin in pubertal children with idiopathic short stature
Source: Endocr Connect. 2018 Apr 18;7(5):708–18. doi: 10.1530/EC-18-0137 (PMC5952247; doi:10.1530/EC-18-0137)
Supplement: Supporting Table 2 [file ec-7-708-t002.pdf]

Supplemental Table 2 Tanner stage of patients treated with GH plus leuprorelin (group A) or GH only (group B), by duration of treatment.

Duration of treatment	Treatment group	Tanner stage, boys, genital score						Tanner stage, girls, breast score					
		n	G1	G2	G3	G4	G5	n	B1	B2	B3	B4	B5
Baseline	A	19	0	11 (57.9)	8 (42.1)	0	0	26	1 (3.8)	17 (65.4)	8 (30.8)	0	0
	B	23	0	16 (69.6)	7 (30.4)	0	0	20	1 (5.0)	13 (65.0)	5 (25.0)	1 (5.0)	0
6 months	A	19	2 (10.5)	10 (52.6)	7 (36.8)	0	0	25	9 (36.0)	13 (52.0)	3 (12.0)	0	0
	B	23	0	7 (30.4)	14 (60.9)	2 (8.7)	0	19	2 (10.5)	6 (31.6)	9 (47.4)	2 (10.5)	0
12 months	A	18	2 (11.1)	10 (55.6)	6 (33.3)	0	0	24	11 (45.8)	10 (41.7)	3 (12.5)	0	0
	B	22	0	4 (18.2)	14 (63.6)	4 (18.2)	0	19	0	4 (21.1)	12 (63.2)	3 (15.8)	0
18 months	A	17	2 (11.8)	8 (47.1)	7 (41.2)	0	0	24	13 (54.2)	7 (29.2)	4 (16.7)	0	0
	B	23	0	0	11 (47.8)	10 (43.5)	2 (8.7)	19	1 (5.3)	0	9 (47.4)	8 (42.1)	1 (5.3)
24 months	A	15	0	9 (60.0)	4 (26.7)	2 (13.3)	0	21	11 (52.4)	7 (33.3)	3 (14.3)	0	0
	B	22	0	0	4 (18.2)	15 (68.2)	3 (13.6)	19	0	0	5 (26.3)	11 (57.9)	3 (15.8)
30 months	A	10	0	3 (30.0)	5 (50.0)	2 (20.0)	0	14	2 (14.3)	3 (21.4)	7 (50.0)	2 (14.3)	0
	B	12	0	0	2 (16.7)	7 (58.3)	3 (25.0)	13	0	0	1 (7.7)	8 (61.5)	4 (30.8)
36 months	A	8	0	0	1 (12.5)	7 (87.5)	0	9	2 (22.2)	1 (11.1)	2 (22.2)	4 (44.4)	0
	B	9	0	0	1 (11.1)	5 (55.6)	3 (33.3)	10	0	0	0	6 (60.0)	4 (40.0)

Data show number of patients (% of total).

n, total number of patients with data available.
